# Complex Ureteral Reconstruction via Open or Robotic Ureteroplasty with a Buccal Mucosa Onlay Graft: A Two-center Comparison

**DOI:** 10.1016/j.euros.2024.11.002

**Published:** 2024-12-17

**Authors:** Antonio Andrea Grosso, Fabrizio Di Maida, Daniele Paganelli, Simon Udo Engelmann, Emily Rinderknecht, Christoph Eckl, Sebastian Kälble, Alexey Barskov, Rino Oriti, Sofia Giudici, Christoph Pickl, Maximilian Burger, Andrea Mari, Andrea Minervini, Roman Mayr

**Affiliations:** a Unit of Oncologic Minimally Invasive Urology and Andrology, Department of Experimental and Clinical Medicine, Careggi Hospital, University of Florence, Florence, Italy; b Department of Urology, St. Josef Medical Center, University of Regensburg, Regensburg, Germany

**Keywords:** Robotics, Reconstructive surgery, Augmented ureteral anastomosis, Partial ureter replacement

## Abstract

**Background and objective:**

Management of a long proximal ureteral stricture is challenging. Buccal mucosal graft (BMG) ureteroplasty is a reliable technique for ureteral reconstruction that avoids the morbidity of bowel interposition or autotransplantation. We compared open and robotic BMG ureteroplasty in a two-center study.

**Methods:**

We compared prospectively recorded data for 26 patients who underwent robotic or open BMG ureteroplasty at two academic institutions. Stricture location and length, previous reconstructive interventions, complications, and success rates were assessed and compared. A descriptive statistical analysis was performed.

**Key findings and limitations:**

We compared ten patients in the robotic group and 16 in the open group. Stricture location had similar distributions in the open versus robotic group (pelvic junction, 25% vs 20%; proximal ureter, 56.3% vs 60%; middle ureter, 18.7% vs 20%). Median stricture length was significantly longer in the robotic group (26 vs 17 mm; *p* = 0.01). The rate of previous reconstructive interventions was higher in the robotic group (80% vs 37.5%; *p* = 0.001). However, previous reconstructive interventions were more complex for the open surgery group. There were no intraoperative complications, and postoperative complication rates were similar in the open and robotic groups (18.7% vs 20%; *p* = 0.19). Median intraoperative blood loss was significantly lower in the robotic group (300 vs 175 ml; *p* = 0.03). The success rate was 93.7% in the open group and 90.0% in robotic group.

**Conclusions and clinical implications:**

We observed high success rates and low perioperative morbidity for both open and robotic BMG ureteroplasty. The robotic approach was associated with significantly lower intraoperative blood loss.

**Patient summary:**

Narrowing of the ureter, which is the tube draining urine from the kidney into the bladder, may need surgical treatment. For reconstruction of long segments, use of a tissue graft from the inside of the mouth is an effective surgical option. Robot-assisted surgery is as safe as open surgery and is associated with lower blood loss.

## Take Home Message

We observed high success rates for open and robotic ureteroplasty with a buccal mucosa graft. The robotic approach was associated with lower intraoperative blood loss.

## 1 Introduction

Management of complex ureteral strictures presents significant challenges from a surgical standpoint, often necessitating intra-abdominal procedures [1]. While distal ureteral strictures may benefit from the Boari flap and psoas hitch techniques, upper ureteral strictures that are unsuitable for ureteroureterostomy or pyeloplasty continue to pose difficulties for urology surgeons [2], [3]. Options such as ileal ureteral replacement and renal autotransplantation are associated with considerable morbidity [4], [5]. Originating from urethral reconstructive surgery [6], buccal mucosal grafts (BMGs) have become increasingly prevalent and have been included in the latest European Association of Urology guidelines as a viable surgical option for ureteral strictures [7]. BMGs are relatively easy to harvest, are compatible with a moist environment and resistant to infection, and have ideal properties for integration in the urinary tract owing to their vascularized epithelium and thin lamina propria [8], [9]. Historically, open ureteral reconstruction has been the standard approach, with good results in this setting [10]. More recently, minimally invasive, and robotic approaches in particular, have become prominent for various urological procedures, including the management of ureteral strictures [11]. Robotic techniques offer technical advantages over open surgery, such as a three-dimensional augmented visual field, easier suturing at various angles and positions, and superior aesthetic outcomes [12]. However, the impact of these advantages on surgical outcomes for this specific technique is unclear, as no comparative study has been published so far. Therefore, our aim was to conduct a retrospective study comparing prospectively collected data for two cohorts undergoing ureteroplasty with BMG at two high-volume centers, one treated with a robotic approach and the other with an open technique.

## 2 Patients and methods

### 2.1 Study design and setting

After institutional review board approval was obtained (Florence: FI-76452; Regensburg: 23-3403-104), data were collected for all patients who underwent robot-assisted ureteroplasty with BMG at Careggi Hospital, University of Florence within a 12-mo period (November 2022 to November 2023). The indication for robotic BMG ureteroplasty was a benign ureteral stricture at the height of the ureteropelvic junction (UPJ) or the proximal or middle ureter that was not amenable to standard surgical reconstruction because of previous reconstructive surgery, stricture length, or peri-ureteral fibrosis. This robotic cohort was then compared to an open surgery cohort of patients treated at Regensburg University Hospital between July 2020 and July 2023 [13].

Demographic data was collected including age, body mass index (BMI) and Charlson comorbidity index (CCI) which was adopted as a proxy of patient comorbidity burden. Cause, location and length of the stricture were also investigated, as well as the type of previous corrective surgery.

Perioperative and postoperative data, including operative time, estimated blood loss (EBL), complications, hospitalization time and readmission rate were accurately recorded. Postoperative complications were graded according to the Clavien-Dindo classification [14]. Follow-up included symptomatic clinical assessment, including evaluation of the surgery site and BMG donor site, as well as ultrasound of the urinary tract. The earliest was performed 3 mo after removal of the ureteral stent and then at 12-mo postoperatively. Success was defined as absence of stricture and ectasia on imaging and the absence of symptoms related to the donor site (difficulties in whistling, reduced saliva production) or surgical site (flank pain) at 3-mo assessment after the double-J stent was removed.

### 2.2 Patient management and surgical technique

Preoperative patient preparation was similar in both centers. All patients received a urinary drainage in the form of a double-J stent if possible and a nephrostomy in cases where double-J stenting could not be performed due to ureteral occlusion. A preoperative retrograde urography was performed, and in cases with a nephrostomy, antegrade urography was supplemented. Postoperatively, double-J stent was removed after 4 wk.

**Fig. 1:**
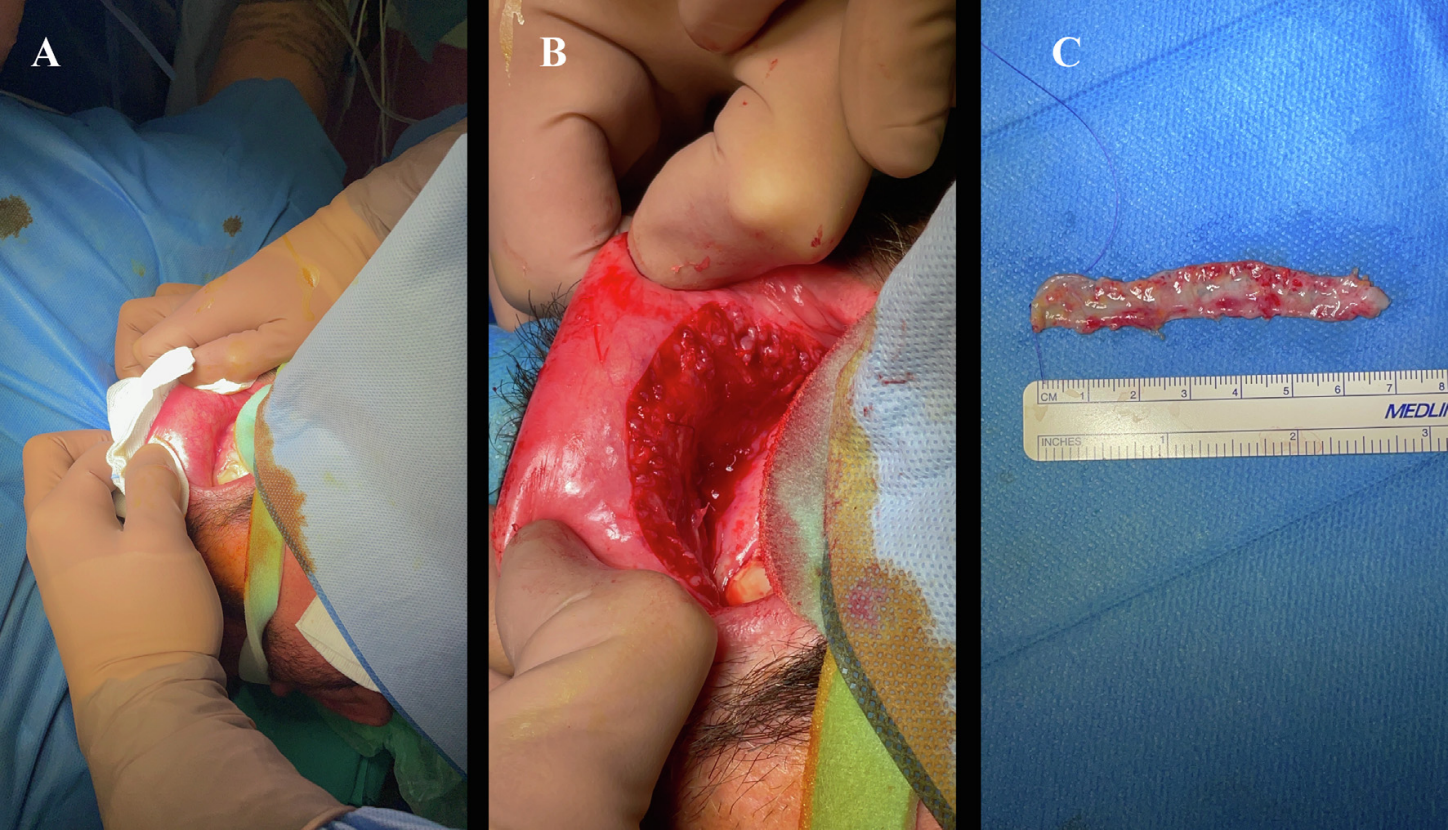
Phases of the harvesting procedure for the buccal mucosal graft.

To facilitate BMG harvesting, all patients underwent general anesthesia via nasotracheal intubation. The graft site on the lower lip was exposed manually by the assistant (Fig. 1). Submucosal saline solution was injected, and precise dissection of the graft margins was carried out with a scalpel. The grafts were standardized at 1.5 cm in width with varying length. Bipolar hemostasis was applied, and the surgical site was closed using 3-0 absorbable sutures. A sterile compress soaked in diluted adrenaline was placed over the wound area and removed before extubation. The graft was then prepared ex vivo by removing submucosal tissue.

**Fig. 2:**
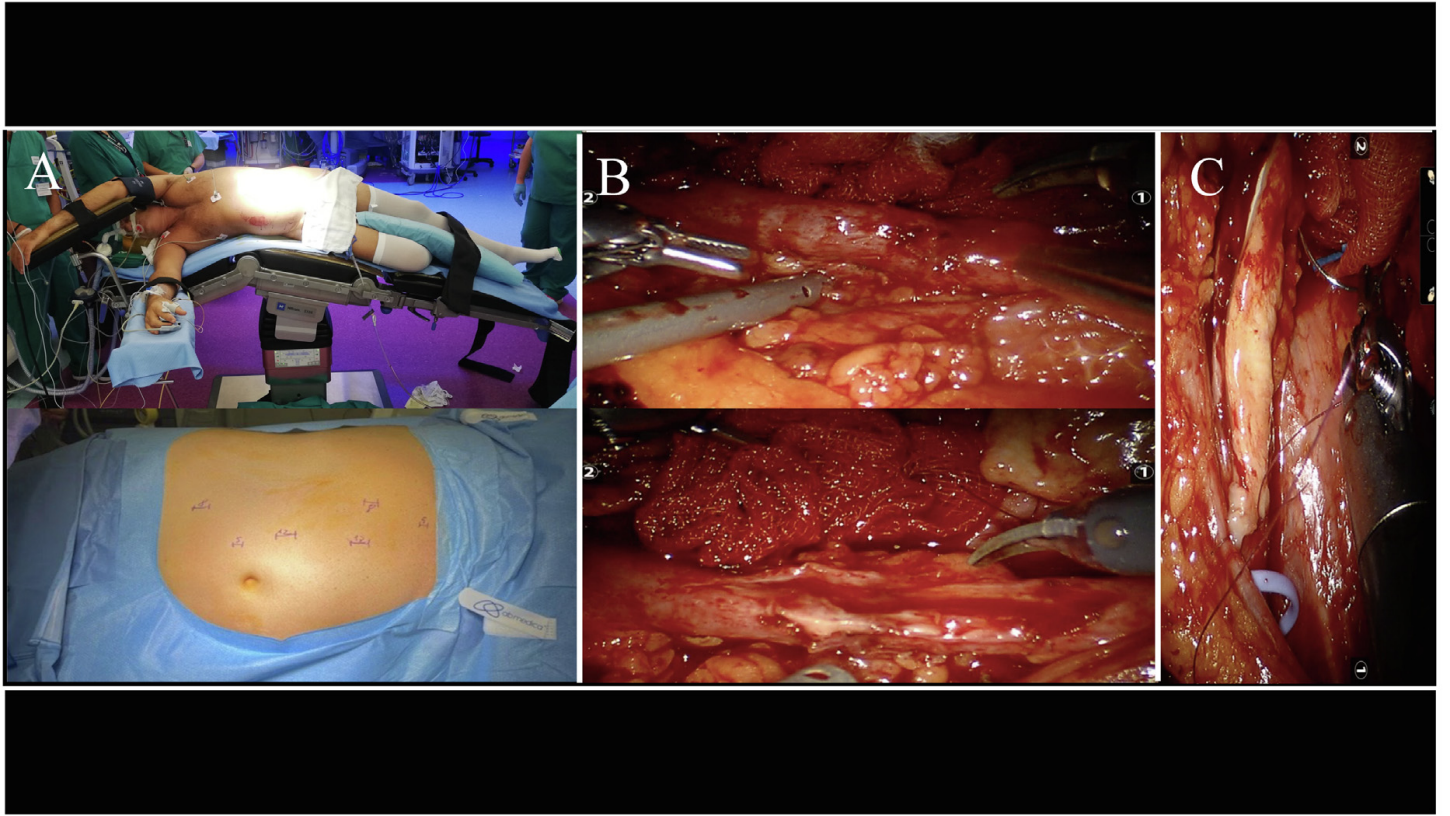
(A) Trocar placement. Surgical phases of the robotic ureteroplasty: (B) isolation and section of the ureter; and (C) anastomosis of the mucosal graft.

Open ureteroplasty was performed using the steps previously described [13]. Robotic surgery was performed with a da Vinci Xi surgical system (Intuitive Surgical, Sunnyvale, CA, USA). Patients were placed in the lateral decubitus position with a breaking angle of 30°. Port placement is depicted in Figure 2A, with slight changes according to the location of the stricture. After identification, the ureter was followed and dissected from adjacent tissue until the stenosis was properly isolated (Fig. 2B). Ureterotomy was performed along the length of the stricture and the BMG was sewn to the ureter edges as an onlay patch over a double-J stent using 5-0 monofilament absorbable running sutures (Fig. 2C). The graft was then covered with an omental flap. A drain tube was placed intra-abdominally and the wound was closed.

### 2.3 Study endpoints

The primary endpoint was evaluation of the safety of BMG ureteroplasty, with comparison of the robotic and open approaches in terms of operative and hospitalization times and complication rates. The secondary endpoint was the success rate of the two approaches.

### 2.4 Statistical analysis

Results are reported as the frequency and proportion for categorical variables, the mean and standard deviation for continuous parametric variables, and the median and interquartile range (IQR) for continuous nonparametric variable. An unpaired T test, Mann-Whitney test, or Pearson’s χ^2^ test was used to compare variables, as appropriate. Statistical analyses were performed using Stata v16 (StataCorp LLC, College Station, TX, USA). All tests were two-sided and statistical significance level was set at *p* < 0.05.

## 3 Results

The study population comprised ten patients in the robotic group and 16 patients in the open group. Baseline patient characteristics are listed in Table 1. There were no significant differences for the open versus robotic groups in median age at surgery (51 yr, IQR 34–62 vs 58 yr, IQR 50–61; *p* = 0.10), median BMI (25.6 kg/m^2^, IQR 23.4–29.2 vs 25.6 kg/m^2^, IQR 23.9–26.6; *p* = 0.07), or median CCI (both 1, IQR 0–1; *p* = 0.24). Lithiasis was the most frequent cause of stricture in both groups (75% vs 80%; *p* = 0.74). Stricture location had similar distributions in the open versus robotic group (UPJ 25% vs 20%; proximal ureter 56.3% vs 60%; middle ureter 18.7% vs 20%). Median stricture length was significantly greater in the robotic group (26 mm, IQR 18–31 vs 17 mm, IQR 7–41; *p* = 0.01). Moreover, robotic ureteroplasty was more frequent as a reintervention after previous corrective surgery (80% vs 37.5%; *p* = 0.001). Previous corrective procedures were predominantly via endoscopy in the robotic group (8/10) and open/laparoscopic/robotic surgery (6/16) in the open cohort.

**Table 1:** Baseline characteristics of patients who underwent open versus robotic ureteroplasty with a buccal mucosal graft

Parameter	Open surgery (*n* = 16)	Robotic surgery (*n* = 10)	*p* value
Median age, yr (IQR)	51 (34–62)	58 (50–61)	0.10
Sex, *n* (%)			0.26
Male	10 (62.5)	4 (40.0)	
Female	6 (37.5)	6 (60.0)	
Median body mass index, kg/m^2^ (IQR)	25.6 (23.4–29.2)	25.6 (23.9–26.6)	0.07
Body mass index category, *n* (%)			
Normal (20–24.9 kg/m^2^)	6 (37.5)	4 (40)	
Overweight (25–29.9 kg/m^2^)	7 (43.8)	6 (60)	
Obese I (30–34.9 kg/m^2^)	2 (12.5)	0 ()	
Obese III (≥40 kg/m^2^)	1 (6.3)	0 ()	
Median Charlson comorbidity index (IQR)	1 (0–1)	1 (0–1)	0.24
Side, *n* (%)			0.42
Right	9 (56.3)	4 (40.0)	
Left	7 (43.8)	6 (60.0)	
Cause of the stricture, *n* (%)			0.74
Lithiasis	12 (43.8)	8 (80.0)	
Iatrogenic (open/laparoscopic/robotic surgery)	3 (18.8)	2 (20.0)	
Malignancy/radiotherapy-induced	1 (6.3)	0 (0.0)	
Unknown	0 (0.0)	0 (0.0)	
Stricture location, *n* (%)			0.21
Ureteropelvic junction	4 (25.0)	2 (20.0)	
Proximal ureter	9 (56.3)	6 (60.0)	
Middle ureter	3 (18.7	2 (20.0)	
Median stricture length, mm (IQR)	17 (7–41)	26 (18–31)	0.01
Previous corrective surgery, *n* (%)			< 0.001
Yes	6 (37.5)	8 (80.0)	
No	10 (62.5)	2 (20.0)	
Type of previous corrective surgery, *n* (%)			<0.001
Endoscopic	0 (0.0)	8 (80.0)	
Open	4 (25.0)	0 (0.0)	
Robotic	2 (12.5)	0 (0.0)	
Preoperative urinary drain, *n* (%)			0.06
Nephrostomy	6 (37.5)	10 (100)	
Double-J stent	10 (62.5)	0 (0.0)	
Median hemoglobin, g/dl (IQR)	13.0 (12.6–14.8)	12.9 (12.6–14.9)	0.22
Median creatinine, mg/dl (IQR)	1.0 (0.86–1.14)	1.0 (0.96–1.32)	0.25
Median graft length, mm (IQR)	50 (50–50)	50 (50–50)	0.18
Median graft width, mm (IQR)	15 (15–15)	15 (15–15)	0.16

IQR = interquartile range.

Perioperative outcomes were comparable for the open versus robotic groups (Table 2) for median overall operative time (154 min, IQR 129–182 vs 165 min, IQR 130–190; *p* = 0.47) and median hospitalization time (both 5 d, IQR 4–5; *p* = 0.22). The open group had higher median EBL (300 ml, IQR 250–350 vs 175 ml, IQR 130–220; *p* = 0.03) and lower median hemoglobin on postoperative day 1 (11.0 g/dl, IQR 10.5–12.8 vs 12.0 g/dl, IQR 11.7–12.9; *p* = 0.05). There were no intraoperative complications, and the postoperative complication rates were similar in the open group (18.7%) and the robotic group (20%; *p* = 0.19). All postoperative complications required hospital readmission, but only one adverse event required surgical management (Clavien-Dindo grade III in the open group), which involved early herniation at the level of the flank incision. There were no infections at the donor site. Finally, there was no significant difference in the rate of success, defined as stricture-free status at the 3-mo assessment, between the open group (93.7%) and the robotic group (90.0%).

**Table 2:** Perioperative outcomes for patients who underwent open versus robotic ureteroplasty with a buccal mucosal graft

	Open surgery (*n* = 16)	Robotic surgery(*n* = 10)	*p* value
Median operative time, min (IQR)	154 (129–182)	165 (130–190)	0.47
Median estimated blood loss, ml (IQR)	300 (250–350)	175 (130–220)	0.03
Median hemoglobin on POD1, g/dl (IQR)	11.0 (10.5–12.8)	12.0 (11.7–12.9)	0.05
Median creatinine on POD1, mg/dl (IQR)	1.02 (0.78–1.14)	1.1 (1.0–1.15)	0.11
Intraoperative complication, *n* (%)	0 (0.0)	0 (0.0)	0.21
Postoperative complication, *n* (%)			0.19
Clavien-Dindo grade ≤II	2 (12.5)	2 (20.0)	
Fever	1 (6.25)	2 (20.0)	
Transfusion	1 (6.25)		
Clavien-Dindo grade ≥III	1 (6.2)	0 (0.0)	
Herniation	1 (6.2)		
Median hospitalization time, d (IQR)	5 (4–5)	5 (4–5)	0.22
Readmission, *n* (%)	3 (18.0)	2 (20.0)	0.11
Median follow-up, mo (IQR)	14 (13–24)	13 (12–14)	0.03
Success rate, *n* (%)	15 (93.7)	9 (90.0)	0.19

IQR = interquartile range; POD1 = postoperative day 1.

## 4 Discussion

BMG ureteroplasty for the treatment of complex upper and middle ureteral strictures remains challenging. For years, the open approach was considered the standard technique, providing success rates ranging from 87.5% to 94.1% according to a 2021 systematic review [10]. Advances in technology and minimally invasive approaches have led to a paradigm change in the surgical treatment of countless urological pathologies [15], [16], [17]. Laparoscopic BMG ureteroplasty was first reported by Li et al [18] in 2015, with successful treatment of one patient. Adoption of laparoscopy was limited by the widespread uptake of robot-assisted surgery, which greatly simplified reconstructive surgeries including ureteroplasty [11]. Here we present the first comparative analysis of open and robotic BMG ureteroplasty cohorts from two referral institutions.

**Fig. 3:**
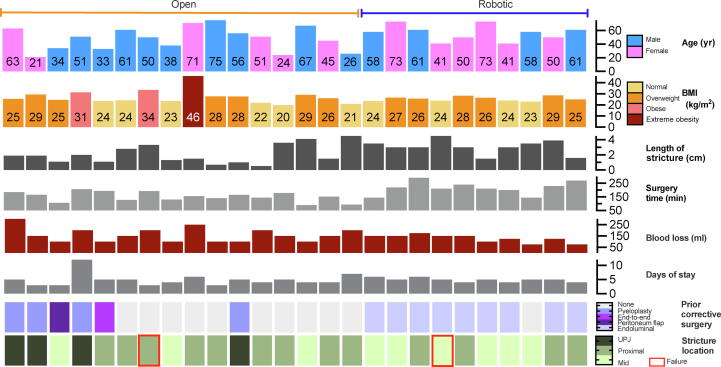
Overall distribution of patient characteristics and surgical outcomes, BMI = body mass index; UPJ = ureteropelvic junction. Figure generated using GraphPad Prism v10.

The primary endpoint was the surgical safety of the two techniques. We found comparable results for operative and hospitalization times and the postoperative complication rate (*p* > 0.05). Only one adverse event following open ureteroplasty required surgical management (Clavien-Dindo grade >II). The only significant difference was for EBL, which was higher in the open group (median 300 vs 175 ml; *p* = 0.03). This translated into lower hemoglobin in the open group on postoperative day 1 (median 11.0 vs 12.0 g/dl; *p* = 0.05); one patient in this group required a blood transfusion. Overall, the two approaches showed a good safety profile, with an acceptable complication rate in both groups. As the robotic approach is less invasive and allows greater accuracy during tissue dissection and thus lower blood loss, we speculate that this strategy may be preferable in older and comorbid patients, especially those with a history of ischemic accidents for whom perioperative bleeding has to be minimized. Several studies have shown the superiority of robotic over open surgery for frail patients for various surgical interventions [19], [20], [21]. Results for the secondary endpoint confirm that both techniques have high efficacy for the treatment of ureteral strictures, as already demonstrated in previous series [22], [23]. We believe that the high success rates in our series (open surgery 93.7%, robotic surgery 90.0%) can be attributed not only to the ideal BMG properties, as previously mentioned [8], [9], but also to the specific surgical nuances used in both approaches. In our series, a ureteral plate was maintained during the open and robotic ureteroplasty procedures, which contributed to revascularization of the anastomosis site. Moreover, the onlay technique with longitudinal incision helps in creating an enlarged ureteral lumen. These factors aid in preventing recurrent stenosis [24]. However, our results should be interpreted in light of some considerations. First, the median ureteral stricture length was longer in the robotic group (26 mm, IQR 18–31) than in the open group (17 mm, IQR 7–41; *p* = 0.01). However, a short stricture can be complex and challenging to reconstruct if a previous open end-to-end anastomosis was performed (patient #5, Fig. 3). Second, 80% of patients in the robotic group and 37.5% in the open group had undergone previous corrective surgery (*p* < 0.001), but none of the previous procedures involved open or robotic surgery in the robotic group, compared to 37.5% in the open group (*p* < 0.001). It is well established that repeat procedures may be burdened by a non-negligible level of difficulty because of extensive tissue reaction and fibrosis [25], [26]. Third, results for the robotic group demonstrate the benefits of robot-assisted surgery in terms of precise and accurate dissection and three-dimension visualization of the surgical field. Finally, other technological tools may be combined with robotic platforms, such as near-infrared fluorescence visualization of indocyanine green, which can provide further intraoperative information on the vascularization of the ureter and the surrounding tissue [27].

Our study has some limitations. In particular, the limited sample size precludes definitive conclusions; the procedures were conducted by different surgeons (R. Mayr for open surgery and A. Minervini for robotic surgery) and despite extensive expertise in their field and the similarity of the procedures, different biases may affect the robustness of the findings. In addition, the short follow-up precludes assessment of the long-term effects.

Despite the limitations, this is the first study comparing two prospectively enrolled patient cohorts undergoing BMG ureteroplasty via an open or robotic approach.

## 5 Conclusions

BMG ureteroplasty is a safe and effective procedure for the treatment of strictures in the upper or middle ureter via either an open or robotic approach, with low complication rates and high success rates in both cohorts. Robotic surgery was associated with significantly lower intraoperative blood loss and a higher postoperative hemoglobin level, with no transfusions required. Further larger studies are warranted to corroborate this evidence.

  ***Author contributions***: Roman Mayr had full access to all the data in the study and takes responsibility for the integrity of the data and the accuracy of the data analysis.

  *Study concept and design*: Grosso, Mayr, Minervini.

*Acquisition of data*: Grosso, Di Maida, Paganelli, Engelmann, Rinderknecht, Kälble, Barskov, Oriti, Giudici, Pickl, Mari.

*Analysis and interpretation of data*: Grosso, Engelmann, Di Maida, Mayr.

*Drafting of the manuscript*: Grosso, Di Maida, Mayr.

*Critical revision of the manuscript for important intellectual content*: All authors.

*Statistical analysis*: Grosso, Di Maida, Engelmann, Mayr.

*Obtaining funding*: None.

*Administrative, technical, or material support*: Kälble, Rinderknecht, Pickl, Barskov, Oriti, Giudici.

*Supervision*: Burger, Di Maida, Minervini.

*Other* (*performed surgery*): Minervini, Mayr.

  ***Financial disclosures:*** Roman Mayr certifies that all conflicts of interest, including specific financial interests and relationships and affiliations relevant to the subject matter or materials discussed in the manuscript (eg, employment/affiliation, grants or funding, consultancies, honoraria, stock ownership or options, expert testimony, royalties, or patents filed, received, or pending), are the following: None.

  ***Funding/Support and role of the sponsor*:** None.
